# Endothelial Progenitor Cells Physiology and Metabolic Plasticity in Brain Angiogenesis and Blood-Brain Barrier Modeling

**DOI:** 10.3389/fphys.2016.00599

**Published:** 2016-12-01

**Authors:** Natalia A. Malinovskaya, Yulia K. Komleva, Vladimir V. Salmin, Andrey V. Morgun, Anton N. Shuvaev, Yulia A. Panina, Elizaveta B. Boitsova, Alla B. Salmina

**Affiliations:** Research Institute of Molecular Medicine & Pathobiochemistry, Krasnoyarsk State Medical University named after Prof. V.F. Voino-YasenetskyKrasnoyarsk, Russia

**Keywords:** endothelial progenitor cells, blood-brain barrier (BBB), neurovascular unit, Angiogenesis, Nicotinamide adenine dinucleotide (NAD+)

## Abstract

Currently, there is a considerable interest to the assessment of blood-brain barrier (BBB) development as a part of cerebral angiogenesis developmental program. Embryonic and adult angiogenesis in the brain is governed by the coordinated activity of endothelial progenitor cells, brain microvascular endothelial cells, and non-endothelial cells contributing to the establishment of the BBB (pericytes, astrocytes, neurons). Metabolic and functional plasticity of endothelial progenitor cells controls their timely recruitment, precise homing to the brain microvessels, and efficient support of brain angiogenesis. Deciphering endothelial progenitor cells physiology would provide novel engineering approaches to establish adequate microfluidically-supported BBB models and brain microphysiological systems for translational studies.

## Endothelial progenitor cells: origin and general characteristics

Neurovascular unit (NVU) consisted of cerebral microvessel endothelial cells, pericytes, astrocytes, neurons, and microglia is a structural and functional basis of the blood-brain barrier (BBB). NVU/BBB is actively involved in the regulation of crucial physiological processes within the brain, but is dramatically compromised in almost all the types of brain pathology (neurodevelopmental disorders, neurodegeneration, trauma, ischemia, neuroinflammation, neuroinfection etc.).

Brain microvascular endothelial cells (BMEC) represent one of the most interesting subpopulations of endothelial cells (EC) due to their specific properties required for the appropriate functioning of these cells: (i) regulation of cerebral blood circulation, (ii) selective and controlled permeability of the blood-brain barrier (BBB). In contrast to endothelial cells of non-cerebral localization, BMEC are characterized by high expression of tight junctions, high electrical resistance, low fenestration, small perivascular space, high prevalence of insulin and transferrin receptors, relatively big number of mitochondria (Stamatovic et al., 2008; Salmina et al., 2014). As it was demonstrated (De Bock et al., 2013), metabolic plasticity of endothelial cells allows rapid switching to active growth upon stimulation, i.e., under the conditions supporting establishment of tip or stalk cells phenotypes with the corresponding proliferative and migratory activity. Thus, metabolism of endothelial cells is considered as a major driver of angiogenesis and might serve as a marker of endothelial dysfunction seen in cardiovascular and cerebrovascular diseases (Eelen et al., 2015). However, another question arises on the metabolic activity of endothelial progenitor cells (EPC) that are responsible for vessel development and (re)endothelialization.

Currently, there is considerable interest to the assessment of BBB-related angiogenesis in developing and adult brain. Development of NVU critically depends on the availability of EPC that originate from embryonic hemangioblasts and hematopoietic stem cells and form the primary vascular plexus (Rae et al., 2011a). Embryonic vasculogenesis and establishment of BBB is driven by newly formed EPC migrating from the sites of hematopoietic stem cells (HSC) development. Bone marrow starts to function as a source of HSC just before birth whereas in embryogenesis, multi-lineage hematopoietic progenitors exist in the extraembryonic yolk sac at E8.25, in the placenta and embryonic aorta-gonad-mesonephros region at E10, and in the fetal liver at E11.0 in mice (Coşkun et al., 2014). Later, in the postnatal brain, EPC may also come from the bone-marrow hematopoietic niches, but their role in the endothelialization, maturation and maintenance of the structural and functional integrity of BBB is not clear yet.

The term “endothelial progenitor cells” initially covered various subsets of circulating progenitors derived from bone-marrow pluripotent stem cells and hemangioblasts. EPC are involved in vessel development and regeneration, being recruited from the bone-marrow to the peripheral blood, displaying immunopositivity for CD34, CD45, CD133, VEGFR2, c-kit, and, presumably existing as hematopoietic cells with pro-angiogenic activity *in vitro* and *in vivo* (Richardson and Yoder, 2011; Yoder, 2012). In adults, there are two origins of EPC: (i) hematopoietic origin (EPC derived from bone marrow multipotent hemangioblasts [VEGFR2(+)VE-cadherin(+)CD45(−) and mesenchymal stem cells (MSC) (CD73(+)CD90(+)CD105(+)CD34(−)CD45(−)]; (ii) and non-hematopoietic origin (EPC found at the sites of extensive angiogenesis but demonstrating no signs of hematopoietic origin, being, probably, derived from tissue multipotent cells) (Chao and Hirschi, 2010; Leeper et al., 2010; Boxall and Jones, 2012).

Bone-marrow-derived MSC possess the ability to migrate though the BBB *in vivo* and *in vitro* models without evident alterations in the barrier's integrity (Matsushita et al., 2011). Interestingly, there are the reciprocal effects of BMEC and MSC in hypoxic conditions: BMEC stimulate differentiation of MSC into EC, whereas MSC stimulate proliferation and migration of BMEC, thereby contributing to local angiogenesis associated with high BBB permeability (Liu et al., 2008).

Brain tissue multipotent cells express markers for mesenchymal stem cells (i.e., CD13, CD105) and pericytes (i.e., PDGFR-β/CD140b, RGS5, Kir 6.1, NG2) and demonstrate strong multilineage potential (Paul et al., 2012). Relative similarity of MSC and pericytes is very well-known. In the adult brain, pericytes originate from the pre-existing pool or from some bone-marrow progenitors, may express wide spectrum of MSC markers in culture (CD44, CD73, CD90, CD105), contribute to the maintenance of BBB integrity being in the tight contact with EC (Pombero et al., 2016). Pericytes and endothelial cells are under the control of perivascular astrocytes that induce their differentiation needed for effective angiogenesis, thereby astroglial dysfunction may affect angiogenesis via dysregulation of EPC/EC/pericytes interactions in cerebral microvessels. In several tissues, aberrant angiogenesis may be caused by the loss of pericytes number and inadequate proliferative response of EC (Ergul et al., 2015).

Thus, vasculogenesis (establishment of new vessels) is provided by EPC differentiated from embryonic hemangioblasts, or adult EPC, multipotent stem cells, and vessel wall-associated mesenchymal-like cells (mesoangioblasts) (Schmidt et al., 2007). Also, adult hemangioblasts have been detected in the CD133+-population of peripheral blood (Loges et al., 2004). Sprouting or splitting angiogenesis (adult vascular growth) is provided by EC pre-existing in the vessel wall, but acquiring new phenotype (tip and stalk cells) and acting with the support of EPC coming from bone marrow or non-hematopoietic sources (Rae et al., 2011b), and pericytes.

In pathological conditions, angiogenesis and vascular remodeling are usually considered as significant components of brain tissue repair program after injury (hypoxic, ischemic, traumatic, inflammatory, toxic etc.), thus, EPC-mediated mechanisms should be of great importance. As an example, in stroke models, mobilization of EPC from bone marrow correlates to the severity of cerebral alterations (Arai et al., 2009). In Alzheimer's type of neurodegeneration, accumulation of amyloid results in excessive angiogenesis and leaky BBB whereas the levels of circulating EPC is dramatically reduced (Lee et al., 2009; Biron et al., 2011). In autism, persistent remodeling of brain microvessels with the characteristics of splitting angiogenesis due to predominant proliferation of pericytes but not EC may affect neuronal connectivity (Azmitia et al., 2016). In diabetes mellitus, cerebral angiogenesis is exclusively enhanced and is associated with the appearance of non-functioning microvessels and decreased ratio of pericytes to EC (Prakash et al., 2012), but hypoxic injury of diabetic brain is characterized by delayed angiogenesis impeding brain tissue repair (Poittevin et al., 2015). In depression, insufficient angiogenesis is caused by the decreased number of EPC in the peripheral blood, low VEGF effects, and elevated levels of anti-angiogenic factors, whereas stimulation of cerebral angiogenesis is a marker of functional recovery (Boldrini et al., 2012; Yamada, 2016). In sum, it is clear that in almost all the types of brain pathology, deficits of circulating EPC attribute to the aberrant angiogenesis, thus suggesting impaired mobilization, migration of these cells to the brain.

Chemokines-, SDF1-, MMP9-, VEGF-, NO-dependent mechanisms are responsible for the mobilization of EPC from the bone marrow, whereas homing of the recruited cells is provided by molecules with high pro-angiogenic potential (i.e., VEGF, IGF, angiopoietins, cytokines, integrins) at the sites of developmental or pathological angiogenesis (Tilling et al., 2009; Caiado et al., 2011). Also, in addition to bone marrow-derived EPC, non-bone marrow-derived cells (tissue resident) could transform to EC and take part in the re-endothelizlization and angiogenesis (Balaji et al., 2013).

According to the current view, circulating EPC may differentiate into EC to restore the endothelial layer, may directly incorporate into injured endothelium, or may secrete pro-angiogenic factors (VEGF, SDF-1, PDGF etc.) or release microparticles to stimulate tip and stalk cells (Li et al., 2015b). Arrival of EPC to the sites of angiogenesis results in the establishment of functional connections between EPC and EC through the coordinated expression of adhesion proteins in a TNFα-dependent manner (Prisco et al., 2015). EPC provide paracrine signaling to facilitate angiogenesis and phenotype changes in the pre-existing resident endothelial (or endothelial progenitor) cells in the tissue-specific manner at the sites of endothelial injury or high metabolic demand (Zhang et al., 2014). Either in embryonic and adult EPC, secretion of pro-angiogenic chemokines is up-regulated by hypoxia simulating in greater expression of CXCR4, CXCR2, and release of CXCL1, CXCL12, macrophage migration inhibitory factor MIF, VEGF. As a result, adhesive capacity of EPC and local tube formation are greatly improved (Kanzler et al., 2013). Thus, EPC whose recruitment from the bone marrow is stimulated by hypoxia or cytokines, serve as cellular carriers of angiogenic regulatory factors instructed to promote angiogenesis or re-endothelialization. Such carrier function of EPC is compromised in aging as it was demonstrated on the reduced secretion of VEGF, IL-8, IL-17, and granulocyte-colony stimulating factor (G-CSF) from elderly human EPC (Kushner et al., 2008).

Recent data suggest that this mechanism seems to be supplemented with the complementary ones. Firstly, there is a transfer of organelles (i.e., mitochondria, lysosomes) through active tunneling nanotubes from circulating EPC [that should be very prone to initiate nanotubes activity similarly to other stem cells (Yang et al., 2016)] to the resident EC (de Cavanagh et al., 2014). Such organelle donation may rescue damaged endothelial cells from apoptosis or, conversely, facilitate active cell death and establishment of a platform for further endothelial replacement. As an example, in the senescent stressed endothelium, lysosomal transfer from EPC improves EC viability, normalizes endothelial-regulated vasorelaxation, and reduces programmed cells death (Yasuda et al., 2011). Secondly, EPC-released membrane-derived microvesicles are responsible for mRNA transfer to EC. To do that, the microvesicles incorporate in EC due to intermolecular interaction of EC proteins with α4 and β1 integrins (Very Late Antigen-4, VLA-4) expressed in microvesicles, thereby promoting EC proliferation, tube formation, and reducing EC apoptosis (Deregibus et al., 2007). It should be mentioned that α4β1 integrins are the receptors for fibronectin and VCAM-1, and the latter is important for BBB functioning in neuroinflammation (O'Carroll et al., 2015).

General population of EPC is very heterogenous and dynamic. Bone marrow hemangioblasts-derived early and late EPC might be determined *in vitro* being different in their ability to form new vessels and to incorporate into growing vascular networks: Late CD31(+)VE-cadherin(+)CD34(+)CD14(−)CD45(−) EPC with high expression of eNOS and VEGFR2 but not early CD31(+)VE-cadherin(−)CD34(−)CD14(+)CD45(+) EPC with low expression of eNOS and VEGFR2 (Fadini et al., 2012; Cheng et al., 2013; Minami et al., 2015). Expression of eNOS is critical for EPC proliferation and migration (Lu et al., 2015) and may contribute to controlling viability of EPC at the sites of induced angiogenesis (Dong et al., 2016). Transcriptomic data confirmed that early EPC are hematopoietic cells with monocytic phenotype and low angiogenic potential, whereas late EPC have greater proliferative potential and high expression of VEGFR2 (Medina et al., 2010) that is ultimately involved in various steps of angiogenesis and is up-regulated in EPC expansion (Smadja et al., 2007). Interestingly, differential expression of VEGFR2 and another universal EPC marker—Tie2—correlates to the ability of EPC to promote angiogenesis: high Tie2 expression is required for re-endothelialization, whereas greater number of low Tie2/high VEGFR2 cells better incorporate into CD31(+) capillaries (Adamcic et al., 2013).

Cell adhesion glycoprotein CD146 is a pan-endothelial marker found in EPC, circulating EC, and in murine brain blood vessels (Schrage et al., 2008; Flores-Nascimento et al., 2015). CD146(+) EPC belong to the group of late EPC with high pro-angiogenic potential. These EPC could be easily distinguished from CD146(+) circulating mature EC: CD146+ CD34+ CD45+ CD133+ or CD117+, and CD146+ CD34+, CD45– CD133– or CD117–, respectively (Delorme et al., 2005). Moreover, proteolytically generated soluble form of this molecule (sCD146) possesses pro-angiogenic activity (Stalin et al., 2013). The exact role of CD146 expressed in cerebral endothelium remains to be evaluated, however, it takes part in the mechanism of lymphocyte extravasation through the BBB in neuroinflammatory conditions (Duan et al., 2013).

Some bone marrow-derived EPC express stem cells marker CD117 (c-kit) (Beaudry et al., 2007) which is involved in MMP9-mediated mobilization of EPC from the bone marrow in a response to high concentrations of VEGF (Heissig et al., 2003), and CXCR4 which is a receptor for CXCL12 chemokine (stromal cell-derived factor-1, SDF-1) involved in the process of EPC mobilization to the peripheral blood. Bone marrow-derived and umbilical cord blood-derived EPC differ in CXCR4 expression even they demonstrate compatible angiogenic properties (Finney et al., 2006).

Increased expression of CXCR4 on EPC is required for adenosine-induced mobilization of these cells from the bone marrow (Rolland-Turner et al., 2013). The remarkable fact is that mature BMEC are also regulated by adenosine which is known as a mediator of BBB structural and functional integrity: acting at A2A adenosine receptors it increases the barrier permeability for some drugs and immune cells for the meaningful time window (Carman et al., 2011; Kim and Bynoe, 2015), therefore being suggested as a potent agent for improving drug delivery to the CNS (Gao et al., 2014). Adenosine in high concentrations is produced in various tissues from ATP by CD39/CD73 ectonucleotidases or, alternatively, via CD38 (or CD157)/CD203a/CD73 pathway sensitive to the local concentrations of NAD^+^ (Horenstein et al., 2013). Bone marrow clonogenic niches are enriched in such enzymatic activities (Quarona et al., 2015), however, it remains to be assessed whether similar mechanism is active at NVU/BBB. At least, very recent hypothesis announced in (Panfoli et al., 2016) stated that elevated levels of adenosine in the brain and blood due to endothelium-driven metabolism of ATP to adenosine in premature infants might be a biomarker of prematurity risk.

Circulating EC might be found in the peripheral blood due to endothelial injury, anoikis, or apoptosis. These cells express the markers of mature differentiating EC (i.e., CD31 and vWF but not CD133), and could also contribute to vascular repair (Tenreiro et al., 2016). Genomic studies revealed that expression patterns of RNAs and miRNAs in EPC and EC are different and reflect significant changes in the functional role of these two types of cells in development and maintenance of endothelium competence (Cheng et al., 2013; Chang et al., 2014).

Pericytes surrounding and supporting endothelial cells share some common properties with EPC (or with bone marrow stromal cells, BMSC): expression of adhesion molecules and VEGFR2, angiopoietin signaling, and prominent pro-angiogenic potential (Bagley et al., 2005; Winkler et al., 2014; Cantoni et al., 2015). That is not surprising due to bone marrow origin of pericytes (Lamagna and Bergers, 2006) and their potential to differentiate into various cell types or to originate from early EPC *in vitro* (Cantoni et al., 2015). Moreover, co-culture of EPC and bone marrow-derived stem cells *in vitro* (mesenchymal stem cells) results in the appearance of pericyte-like cells due to ability of bone marrow-derived stem cells to differentiate into pericytes with the paracrine regulatory activity of EPC (Loibl et al., 2014). In the context of BBB, pericytes origin and functioning are of great importance due to higher (10–30-fold) pericyte/endothelial cell ratio in the central nervous system (CNS) comparing to other tissues (Winkler et al., 2014).

Aberrant physiology of EPC is tightly implicated in the pathogenesis of various types of pathologies associated with endothelial dysfunction and vasculopathy including diabetes mellitus, Alzheimer's disease, cerebrovascular and cardiovascular diseases etc. (Lee et al., 2010; Brea et al., 2011; Yiu and Tse, 2014; Berezin and Kremzer, 2015). Circulating CD146(+) EPC as well as BMEC have been proposed as novel biomarkers of BBB impairment in neuroinfection and neurotoxicity (Huang et al., 2013a) applicable for diagnostic purposes. At the same time, there is a growing evidence that EPC might serve as a therapeutic tool for a number of CNS pathologies including stroke, neurodevelopmental disorders, and neurodegeneration (Castillo-Melendez et al., 2013; Fukuda et al., 2013; Zhao et al., 2013). Very promising data have been obtained in the application of EPC for BBB repair *in vivo* (Huang et al., 2013b). However, application of EPC into routine neurological clinical practice is hampered by poor understanding the mechanisms underlying EPC homing at CNS and EPC-mediated cell-to-cell communications in cerebral microvessels within the NVU.

Migration of EPC to the BBB is dictated by numerous factors contributing to elevated permeability of BBB that might serve as signals for EPC mobilization and moving toward the brain tissue. Studying brain angiogenesis and BBB establishment and maturation (so-called barriergenesis) offers some unique opportunities to distinguish a role for EPC in either developmental or pathological angiogenesis since both these processes have different mechanisms and functional significance for the developing, mature and aging brain (Vallon et al., 2014). Metabolic activity of EPC and related cells with pro-angiogenic potential is not well-studied yet. There is no doubt that metabolism of EPC and metabolic microenvironment at the sites of their destination affect efficacy of angiogenesis. As an example, remodeling of primary capillary plexus and vasculogenesis during embryogenesis depend on the actual metabolic demands of the defined tissue (Yoder, 2012). Particularly, EPC metabolic activity in the resting state and upon stimulatory conditions (i.e., expansion in the bone marrow, mobilization, and targeted migration) differs. The same should be true for BMEC activated in angiogenesis and converted to tip or stalk phenotype.

**Figure 1:**
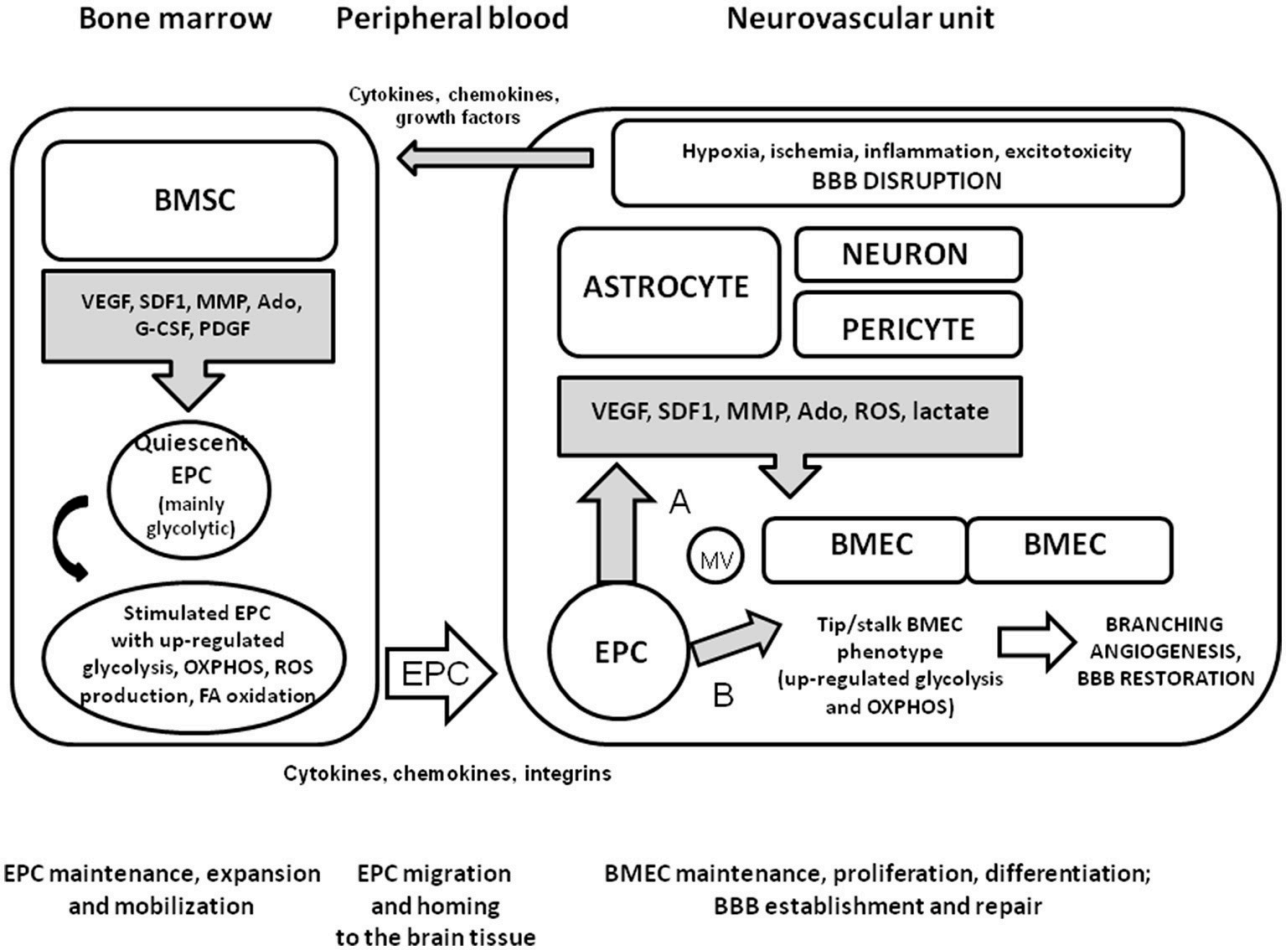
**Mobilization and homing of EPC to the injured brain tissue**. Within the NVU, pericytes and perivascular astrocytes produce the molecules with pro-angiogenic properties upon neuronal overexcitation, hypoxic or ischemic brain injury, BBB dysfunction, or neuroinflammation. Bone marrow stromal cells secrete various factors contributing to maintenance or expansion of EPC when needed. In a quiescent state, EPC have high glycolytic activity due to relatively hypoxic micronevironment within the clonogenic niche. Being activated by cytokines, chemokines, growth factors whose systemic and local concentrations are elevated due to brain injury, EPC up-regulate metabolic pathways for effective energy production (glycolysis, mitochondrial respiration, fatty acid oxidation). Generation of ROS is enhanced as a side-effect of mitochondria activation, but is counteracted by well-established antioxidant machinery in EPC. Homing of recruited EPC to cerebral microvessels is driven by cytokine-, chemokine-, and integrin-based mechanisms. Upon arrival at the site of BBB disruption, EPC release pro-angiogenic factors and membrane vesicles enriched with EPC-specific proteins and mRNA (A); incorporate into the endothelial layer or donate organelles to the stressed EC (B). These mechanisms lead to the stimulation of branching angiogenesis associated with the activation of tip and stalk EC, and re-establishment of BBB.

Thus, it is reasonable to propose that local microenvironment in the bone marrow supporting EPC maintenance, expansion, and mobilization would have some similarities with the microenvironment providing within the NVU to support BMEC functional activity, BBB establishment and repair. Since elevated permeability of BBB is associated with the establishment of sites of active neurogenesis (Lin et al., 2015b), migration of EPC from the hematopoietic tissue to the adult BBB is a transfer from one clonogenic niche to another one. Upon coming to the brain microvessels, EPC incorporate in the endothelial layer or serve as a source of pro-angiogenic molecules in a way similar to pericytes and perivascular astrocytes, thereby establishing appropriate conditions for branching angiogenesis, restoration of BBB structural and functional integrity, and reparative neurogenesis (Figure 1).

**Figure 2:**
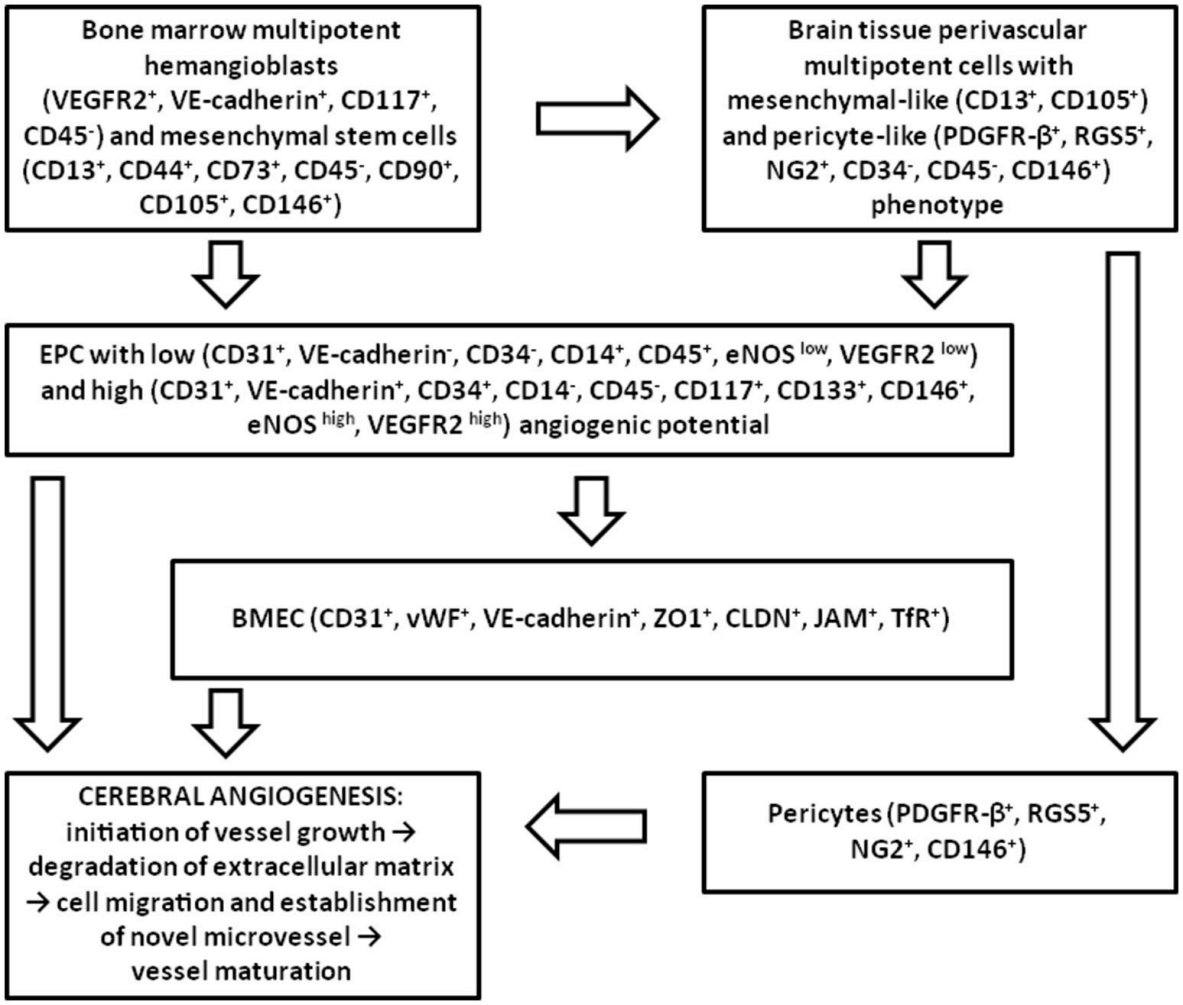
**Participation of different types of EPC in adult brain angiogenesis**. In the adult brain, EPC originated from the bone marrow multipotent hemangioblasts and MSC, or from the multipotent mesenchymal-like and pericyte-like cells located in cerebral microvessels, give rise to the population of EPC which is able to activate BMEC, to integrate into the endothelial layer, and to promote recruitment and proliferation of pericytes upon the action of pro-angiogenic stimuli. Later, perivascular cells coordinate acquisition of endothelial quiescence and vessel maturation.

Angiogenesis consists of cell proliferation, vessel sprouting, establishment of anastomosis, pruning and remodeling, acquisition of endothelial quiescence (Ehling et al., 2013). Antigenic and functional heterogeneity of endothelial progenitors determines the mechanisms of EPC-supported angiogenesis. In the brain, vascularization mainly occurs through angiogenesis (Grant and Janigro, 2006), therefore different populations of cells involved into angiogenic events contribute to the initiation of vessel growth, destabilization of extracellular matrix, establishment of novel microvessels and their maturation (Figure 2). In the context of BBB, the latter stage is corresponded to the maturation of the barrier and acquisition of adequate selective permeability and transporting activity. Presumably, all the earlier steps of angiogenesis are coupled with the elevated permeability of the BBB. Therefore, a reasonable question is how all these processes are linked to the metabolic plasticity of endothelial progenitors and other cellular components of the NVU (neurons and glia)?

## Oxidative metabolism of progenitor endothelial cells and metabolic status of brain tissue: complementary features

Developmental angiogenesis and, probably, neuroplasticity-associated angiogenesis, are mainly regulated by local production of pro-angiogenic molecules (VEGF, TGFβ) and corresponding changes in the metabolism, proliferation, and differentiation status of migrating EPC governed by Wnt/β-catenin- and Notch-signaling (Vallon et al., 2014). In pathological angiogenesis associated with brain injury, the main stimuli provoking re-endothelialization are neuroinflammatory mediators (i.e., cytokines, growth factors) as well as hypoxia/ischemia-induced changes in the tissue metabolism that mobilize EPC from bone marrow and attract them to the brain tissue. As an example, membrane-bound Kit-ligand expressing on microvascular EC at the sites of inflammation provide effective homing of EPC to the activated endothelium (Dentelli et al., 2007).

It is reasonable to assume that neuroplasticity-associated angiogenesis might critically depend on the local branching provided by pre-existing BMEC acquiring tip or stalk phenotype, whereas developmental and pathological angiogenesis would require extensive mobilization and homing of bone marrow-derived endothelial progenitors. It is still under the debates which local metabolic factors in the brain are attributed to homing of EPC to the brain and their integration into the developing cerebral microvessels, or how it is corresponded to the metabolic pattern of EPC.

### Metabolic plasticity of EPC

One of the most potent local regulators of angiogenesis is hypoxia and associated metabolic events, particularly, stimulation of glycolysis. At the same time, lactate as the end-product of glycolysis has multiple functions in the brain being involved in the coordination of neuron-astrocyte metabolic coupling and gliovascular control of local blood flow (Mosienko et al., 2015; Kasparov, 2016). There is an accumulating evidence for glycolysis-mediated control of BBB development and functional activity (Salmina et al., 2015). EPC like other stem/progenitor cells are characterized by active glycolysis and high production of lactate (Goligorsky, 2014). When resident tissue BMEC acquire tip cell phenotype, glycolysis is also intensified (Stapor et al., 2014). In the BBB, expression of endothelial MCT1 transporting ketone bodies, pyruvate, and lactate is maximal in the perinatal period followed by dramatic decrease in first 1 week of postnatal life with the corresponding switch to prevailed expression of GLUT on endothelial cells and MCT1 on astroglial cells at the postweaning period in rodents (Vannucci and Simpson, 2003). Such developmental changes reflect predominant consumption of lactate and ketone bodies or glucose in fueling the developing and maturing brain, respectively. Thus, EPC migrating to cerebral microvessels in perinatal and early postnatal period appear in the microenvironment of excessive lactate and ketone bodies utilization. However, such proposal requires further assessment since recent transcriptome study has shown that MCT1 is expressed at lower levels in the embryonic choroid plexus (E15) than in the adult brain, whereas GLUT1 is expressed in an opposite manner (Saunders et al., 2013, 2015).

High-energy metabolites lactate and ketone bodies are known as promotors of stem cells or cancer cells growth and best “mitochondrial fuels” for actively-proliferating cells. To achieve such effects of lactate and ketone bodies, the target cells should express MCT1 for effective import of these metabolites and their utilization in the intracellular pathways (Curry et al., 2013). One of the possible explanations of this phenomenon was already given (Martinez-Outschoorn et al., 2011): both these metabolites increase the pool of acetyl-CoA, leading to increased histone acetylation and elevated gene expression. These authors suggested that since the brain is a particularly lactate-rich microenvironment due to lactate-producing activity of astrocytes in a close vicinity to active neurons (so-called astrocyte-neuron metabolic coupling), metastatic cancer cells may be attracted to the lactate-rich microenvironment of the brain. Similar reasoning shows that this might be true for EPC migrating to developing BBB where lactate and ketone bodies levels are rather high due to extensive expression of their transporters at the endothelial layer and great dependence of developing brain on these energy substrates. Taking into the consideration the enrichment of BBB endothelial cells with mitochondria comparing to EC in other tissues (Oldendorf et al., 1977), and obvious coupling of mitochondrial biogenesis to angiogenesis (De Bock et al., 2013), it is reasonably to propose that high expression of MCT1 in endothelial BBB cells as well as activation of astrocytic glycolysis and release of lactate at the prenatal and early postnatal period would provide the microenvironment adequate for homing and local proliferation of EPC. It is interesting that EPC as well as mature EC demonstrate the compatible rate of proliferative activity *in vitro*, and the number of mitochondria which is less in EPC at the beginning of cell culture is very rapidly elevated to support proliferative activity (Rae et al., 2011a).

Utilization of ketone bodies leads to elevating acetyl-CoA and depleting NAD+ levels in the cells (Newman and Verdin, 2014). Thus, availability of NAD+ for the activity of NAD+-converting enzymes like NAD+-glycohydrolases (polyADP-ribosyl polymerase, ADP-ribosyl cyclase, monoADP-ribosyl transferases) or NAD+-dependent deacetylases (sirtuins) is reduced. The latter might result in higher levels of histone acetylation and maintenance of transcriptionally active chromatin in the cells.

Thus, EPC and newly-formed BMEC in the embryonic or early postnatal BBB might be characterized by consuming of ketone bodies and lactate, considerable mitochondrial activity, intracellular depletion of NAD+, suppression of histone deacetylation, and activation of gene transcription. In accordance with this metabolic pattern, we may propose that when BBB starts expression of GLUT for predominant consumption of glucose for NVU metabolic needs, glycolytic flux is activated as much as possible in BMEC, NAD+ is effectively regenerated due to pyruvate to lactate conversion, and NAD+ is easily used by NAD+-glycohydrolases or stimulates deacetylase activity. Probable involvement of NAD+-glycohydrolases (i.e., CD38) abundantly expressed in activated astroglial cells (Banerjee et al., 2008; Salmina et al., 2009) in the establishment of pro-angiogenic brain microenvironment might be suggested by analogy with the mechanism recently proposed in (Horenstein et al., 2015).

Notch signaling is one of the key regulators of oxidative cell metabolism: high proliferative potential of tumor cells corresponds to hyperactive Notch, suppression of glycolysis and preserved mitochondrial activity, whereas low proliferative potential is attributed to hypoactive Notch, suppressed glycolysis and attenuated mitochondrial respiration (Landor et al., 2011). During embryogenesis, Wnt/β-catenin up-regulates Dll4 transcription and strongly increases Notch signaling in the endothelium, whereas excessive Notch activity results in vascular abnormalities (Corada et al., 2010). Notch signaling is a key regulator of vessel maturation and quiescence in the retinal vasculature: Dll4-Notch signaling supports the quiescent endothelial phenotype up to P28 (Ehling et al., 2013), promotes stalk phenotype of EC and results in less number of tip cells and fewer vessel branches. As expected, inhibition of Notch (i.e., with a gamma-secretase inhibitor) stimulates angiogenesis (Hellström et al., 2007). In endothelial cells, diminished Notch activity due to sirtuin-mediated deacetylation results in excessive vessel branching (Guarani et al., 2011). Whether analogous mechanism is true for EPC remains to be assessed but some data might confirm this proposal: Proteomic and functional analysis has revealed that Notch signaling is required for EPC functioning and angiogenesis (Karcher et al., 2015), but slight reduction of Notch signaling promotes EPC activity and reduces EPC apoptosis (Ii et al., 2010). Presumably, this bidirectional effect is linked to EPC heterogeneity: Notch signaling regulates EPC functions differentially in early (stimulation of cell proliferation, migration) and late—more matured—EPC (suppression of cell proliferation, migration, and vessel sprouting) (Chen et al., 2012).

In actively proliferating cells, Wnt-signaling serves as a positive regulator of glycolysis (Pate et al., 2014). It should be mentioned that activation of Wnt/β-catenin signaling pathway in mature brain endothelial cells results in up-regulation of MCT1 expression, whereas inhibition of gamma-secretase and corresponding suppression of Notch signaling reduces Wnt/β-catenin effects on MCT1 expression (Liu et al., 2016). So, MCT1 expression in BMEC critically depends on Wnt/β-catenin- and Notch-signaling, and the same is true for GLUT1 and claudin-5 expression in the BBB (Vallon et al., 2014). Among other regulators of MCT1 in BMEC are cAMP-generating intracellular signaling pathways (cAMP induces phosphorylation and internalization of MCT1 proteins) (Smith et al., 2012) and intracellular pH (acidic pH inhibits while alkaline pH activates MCT1 activity) (Uhernik et al., 2011). The same Wnt-signaling pathway is critical for the generation of EPC from pluripotent stem cells (Lian et al., 2014) that might be under the control of oxygen availability and HIF-1 activity in undifferentiated cells: low oxygen concentrations in clonogenic niches result in the stabilization of HIF-1 followed by the activation of Wnt-signaling (Mazumdar et al., 2010; De Miguel et al., 2015). Thus, it is not surprising that next step in the development of EPC into EC is controlled by Wnt-signaling as well: over-expression of HIF-1 in EPC promotes EPC proliferation, migration, and differentiation to EC with clearly distinguishable endothelial phenotype CD31(+)VEGFR2(+)eNOS(+) (Jiang et al., 2008). Therefore, high proliferative and pro-angiogenic potential of EPC is equivalent to high HIF-1 activity (due to relative oxygen deficits in clonogenic niches either in bone marrow or in the developing brain), activated Wnt-signaling, and prominent MCT1 expression in these cells.

Actually, this mechanism seems to be very similar to the so-called “reverse Warburg effect” proposed for some tumor cells. It is well-known that Warburg effect is driven by the modulation of Wnt-signaling: suppression of Wnt leads to reduced glycolytic flux, and MCT1 appears to be one of the targets (Pate et al., 2014). This mechanism helps the cells to direct pyruvate from TCA to LDH-mediated conversion to lactate followed by its release from the cells. Similar mechanism has been proposed as a basis for tumor-directed establishment of microenvironment supporting tumor growth. According to this idea, tumor cells make the surrounding stromal or endothelial cells more glycolytic via stabilization of HIF-1 for effective generation of lactate (and, probably, ketones and pyruvate) and its export for feeding the tumor cells equipped with MCT1. The same effect has been attributed to the mechanisms of tumor neoangiogenesis: Tumor cells stimulate endothelial cell migration, tube formation, and tumor angiogenesis through the induction of HIF-1 in endothelial cells (Doherty and Cleveland, 2013) due to HIF-1α stabilizing activity of released lactate (De Saedeleer et al., 2012). In the developing BBB, the main source of lactate is astroglia surrounding endothelial layer (Salmina et al., 2015), therefore establishment of high local concentrations of lactate may provide the microenvironment optimal for EPC migration and EPC differentiation toward BMEC. Taking into the consideration the stimulatory effect of lactate on EPC mobilization *in vitro* (Milovanova et al., 2008a), we may assume that high local lactate concentrations established in bone marrow or in the perivascular space of BBB are the prerequisite for effective participation of EPC in brain angiogenesis.

Release and utilization of lactate is coordinated by MCT expression which is a target for CD147-mediated control. CD147 (extracellular matrix metalloproteinase inducer EMMPRIN, or basigin) is one of the well-known inducers of angiogenesis contributing to the following processes: (i) lactate utilization in the cells due to action of CD147 as a chaperone for MCT1 and MCT4 lactate transporters to facilitate their membrane expression; (ii) glucose uptake through CD147 interaction with GLUT1; (iii) amino acid transmembrane transport due to functional association of CD147 with CD98 (Xu and Hemler, 2005; Muramatsu, 2016). Role of CD147 in angiogenesis is further confirmed by its interactions and stimulatory action on MMP and VEGFR2 (Bougatef et al., 2009) that might have an importance for the recruitment of responding subpopulation from the bone marrow (Chen et al., 2015). It was recently shown that cyclophilin A which is one of CD147 ligands in various tissues acts on CD117 (c-kit)–immunopositive bone-marrow progenitors, thereby contributing to angiogenesis (Perrucci et al., 2016). Proteomic analysis of EPC revealed expression of CD147 protein (Kaczorowski et al., 2013). On other hand, CD147 is highly expressed in the capillary endothelium in the CNS being known as neurothelin for a long time (Kaushik et al., 2015). Moreover, it was proposed as an earliest molecular marker for endothelial cells that will form the blood-brain barrier (Schlosshauer and Herzog, 1990) whose expression is positively regulated by neighboring astroglia (Janzer et al., 1993). This may seem paradoxical that recent “renaissance” in CD147 studies mainly relates to tumor cells oxidative metabolism or tumor-induced angiogenesis, but not to the role of CD147 in the BBB. In sum, it is evidently necessary to assess whether CD147 expressed in EPC and BMEC might serve as a regulator of local pro-angiogenic microenvironment within the bone marrow and the BBB.

A shift in perspective is needed when we are talking about biological role of glycolysis in progenitor cells. It is clear that glycolytic activity determines not only lactic acid production but is also responsible for maintaining NAD+/NADH ratio due to pyruvate-lactate conversion at the final step of the process. According to this view, NAD+ regeneration at this stage contributes to intracellular NAD+ pool, thereby providing indirect regulatory action on NAD+-consuming (i.e., NAD+-glycohydrolases) and NAD+-dependent (i.e., sirtuins) mechanisms (Salmina et al., 2012).

Stem and progenitor cells seem to be critically depended on NAD+ levels, and NAD+ deficit results in loss of self-renewal capacity or impairment of differentiation. Many metabolic processes are very sensitive to changes in NAD+ bioavailability, i.e., chromatin acetylation/deacetylation, oxidative metabolism, intracellular calcium mobilization, and calcium-dependent processes (migration, adhesion, programmed cell death etc.). We may apply those considerations to some of the mechanisms linking NAD+ and functional activity of EPC as shown below.

### NAD+ levels and progenitor cells functioning

Mobilization of EPC from the bone marrow *in vivo*, their migration, proliferation and angiogenic activity *in vitro* can be inhibited by suppressing the activity of nicotinamide phosphoribosyltransferase (NAMPT) which is the key enzyme for NAD+ synthesis, whereas overexpression of NAMPT led to a SIRT1-depedent enhancement of Notch-1 intracellular domain (NICD) deacetylation, inhibition of Notch signaling, up-regulation of VEGFR2 and VEGFR3 expression, and neovascularization (Wang et al., 2014). Depletion of intracellular NAD+ levels is associated with EPC impairment in diabetic patients, whereas restoring NAD+ pool rescued EPC mobilization due to stromal cell-derived factor-1α (SDF-1α)-mediated events and eNOS expression in EPC (Wang et al., 2016). So, it is quite clear that effective angiogenesis requires high intracellular NAD+ levels in EPC and, presumably, in BMEC. However, very recent data suggest that in some tissues, NAD+ synthesis might be positively regulated by the proliferator-activated receptor gamma coactivator-1α (PGC-1α) (Tran et al., 2016). At the same time, PGC-1α is a well-described regulator of mitochondrial biogenesis due to activation of PPARγ and a wide spectrum of transcription factors. In a case of angiogenesis demand, EPC in the peripheral blood as well as endothelial cells in tissue elevate expression of PGC-1α resulting in the activation of Notch signaling and suppression of EPC migration, inhibition of angiogenesis and re-endothelialization (Sawada et al., 2014). Nevertheless, there is no contradiction here, because intracellular NAD+ levels represent very labile and compartment-specific parameter. Therefore, it is reasonably that dynamic changes in NAD+ bioavailability due to glycolytic flux, mitochondrial activity and utilization of NAD+ as a substrate for enzymatic conversion differentially affect SIRT1/Notch-machinery in EPC. In addition, as we discussed above, NAD+-sensitive pathways (i.e., local production of adenosine in the bone marrow or, presumably, at the BBB) might contribute to the regulation of EPC fate and BMEC activity.

### NAD+ levels and progenitor cells senescence

Stem/progenitor cells in general, and EPC, particularly, undergo process of cellular senescence being subjected to inappropriate conditions triggering accumulation of genetic defects, inactivation of telomerase activity, pro-apoptotic changes, depletion of total and mitochondrial NAD+ pools, whereas restoration of NAD+ levels postpones senescence-associated changes (Zhu et al., 2006; Kushner et al., 2011; Son et al., 2016; Zhang et al., 2016).

AMP-activated protein kinase (AMPK) appears to be a good candidate for linking oxidative metabolism and NAD+ levels in EPC. Upon activation, AMPK promotes ATP-generating processes (i.e., glycolysis, fatty acid oxidation) and inhibits ATP-consuming processes (i.e., biosynthesis). In general, AMPK activity results in elevating the intracellular NAD+ levels (Cantó et al., 2009). In relation to foregoing effects of CD147 on EPC, we should mention that AMPK activity is in the tight functional connection with CD147-CD98hc complex in actively proliferating epithelial cells: loosening CD147-CD98hc complex leads to the suppression of cell proliferation and activation of AMPK (Xu and Hemler, 2005).

Recent data reveal pro-angiogenic effect of AMPK activator cilostazol either in EPC or EC (Tseng et al., 2016). It is interesting to mention that activation of AMPK and sirtuin 1 might be also achieved by the anti-diabetic drug metformin, caloric restriction, physical exercise, or resveratrol. The latter one induces NAD+- and PGC-1α-dependent enhancement of mitochondrial function and mitochondrial biogenesis in muscle cells (Price et al., 2012). Experimental data suggest that this AMPK-related mechanism might be, at least partially, responsible for well-known numerous positive effects of resveratrol on EPC proliferation, differentiation, their contribution to angiogenesis, and prevention of cellular senescence due to telomerase activation (Wang et al., 2007; Xia et al., 2008; Campagnolo et al., 2015), pro-reparative effect of physical exercise on EPC mobilization (Kazmierski et al., 2015), and metformin-induced normalization of EPC differentiation (Li et al., 2015a). Related mechanism seems to be actual for EC derived from induced pluripotent stem cells (Jiang et al., 2015). In the context of BBB, overexpression of AMPK in BMEC preserves the structural and functional integrity of the barrier in neuroinfection (Zhao et al., 2014). The same is true for the effect of resveratrol on BBB in neuroinflammation *in vitro* (Hu and Liu, 2016), or for the effect of metformin on BBB in ischemic brain *in vivo* (Liu et al., 2014). But it remains unclear how AMPK activity might contribute to EPC homing at CNS, and barriergenesis. Moreover, in the more complicated multicellular systems, effects of AMPK activators might be quite different as it follows from the recent data on both positive and negative (time of application-dependent) effects of AMPK activation on neuronal survival in neonatal hypoxic-ischemic brain injury (Rousset et al., 2015) This finding emphasizes the importance of evaluating the presumable neuroprotective activity of any drug-candidate in ontogenetic aspect and in the context of cell-to-cell communications within the NVU.

Table 1 summarizes general metabolic characteristics of EPC in comparison to EC and BMEC.

**Table 1:** **Key metabolic properties of EPC, EC/BMEC**.

	**EPC**	**EC, BMEC**
Basal glycolytic rate and lactate production	High	High, particularly in phalanx and stalk cells
Glycolytic rate and lactate production upon stimulatory conditions (expansion, mobilization, migration)	Elevated (not more than 2-fold)	Elevated in tip and stalk cells, suppressed when branching is reduced
Mitochondrial number and OXPHOS intensity	Low mitochondrial mass, immature mitochondrial morphology; low OXPHOS	High mitochondrial mass in BMEC comparing to EC in other tissues; OXPHOS is less notable than glycolysis
Mitochondrial number and OXPHOS intensity upon stimulatory conditions (expansion, mobilization, migration)	Up-regulated	Up-regulated
Mitochondrial ROS production upon basal and stimulatory conditions (expansion, mobilization, migration)	Up-regulated upon EPC stimulation but the antioxidant activity is high	Low in quiescent cells but up-regulated in branching angiogenesis
Utilization of ketone bodies	High	High at the earliest stages of ontogenesis
Fatty acid oxidation	Relatively low, elevated in stimulatory conditions	High, particularly in low glucose conditions
Pentose phosphate pathways activity	Low	High
Lactate-mediated effects	Stimulates migration and differentiation	Stimulates angiogenesis
Physiological and biochemical heterogeneity	High	Low

Based on the integrated data presented in Oldendorf et al., 1977; Dernbach et al., 2004; Milovanova et al., 2008b; Fraisl et al., 2009; Freeman and Keller, 2012; De Bock et al., 2013; Goligorsky, 2014; Harjes et al., 2014; Tang et al., 2014; Xu et al., 2014; Salmina et al., 2015; Schoors et al., 2015.

## Microphysiological systems and microfluidics-based BBB models *in vitro*: application of EPC

Development of relevant blood-brain models *in vitro* is one of the topic problems in modern neurobiology and neuropharmacology. The “ideal” model should match the following criteria: (i) combination of minimally required cell types critical for structural and functional BBB integrity; (ii) reconstruction of the key mechanisms responsible for selective permeability of the BBB; (iii) long-term preservation of functional and structural integrity during the model culturing *in vitro*; (iv) reproduction of specific properties of the defined cell population within the model in (patho)physiological conditions; (v) relative simplicity of assembling the barrier and assessment of its permeability.

Among all the approaches proposed to achieve the above-mentioned goals, using the stem cell-derived components of the BBB in the models *in vitro* appears as very promising solution, particularly, for some special tasks (i.e., modeling the developing BBB, assessment of BBB permeability corresponding to the neonatal period etc.). This direction is further actualized while thinking about microphysiological systems reflecting basic properties of biological tissues and organs in the miniature scale.

It is now possible to see the increasing significance of the knowledge of EPC contribution to BBB development and functioning in the context of microphysiological systems (MPS). MPS are the complex *in vitro* models of tissues and organs (combination of so-called “organoids”) aimed to establish their functional interrelations. In most the cases, it is the next step after the application of microfluidic technologies to the reconstruction of “organ-on-chip” or “body-on-chip” in the microenvironment close to the real one. Moreover, “physiome-on-chip” concept (Stokes et al., 2015) could be more beneficial in obtaining relevant physiological data using MPS approach.

It should be mentioned that microfluidics has influenced BBB modeling *in vitro* a lot: several successful attempts to establish functional BBB on the microfluidic platform have been reported (Booth and Kim, 2012; Griep et al., 2013; Cho et al., 2015). At the same time, there is also increasing interest in the utilization of stem cells-derived material for BBB constructing *in vitro*: development of astrocytes and neurons from neural progenitor cells (Lippmann et al., 2011; Khilazheva et al., 2015), development of BMEC from induced pluripotent stem cells (Lippmann et al., 2012), or even establishment of the barrier from BMEC and neural stem cells *in vitro* (Chou et al., 2014).

Complementary properties of both these approaches will make possible the reconstruction of stem cells-derived cell components of NVU/BBB in the improved microenvironment like it was recently suggested for “brain-on-chip” technologies (Alcendor et al., 2013; van der Helm et al., 2016) applicable for effective *in vitro* drug screening. In this context, reconstruction of brain microvasculature in a flow-directed MPS is one of the topic question which might be solved using EPC as not only a source of BMEC, but also as informative monitoring system to study angiogenesis/barriergenesis “on-line.”

So, what are the possible advantages in the application of EPC for NVU/BBB modeling *in vitro* and MPS designing? Establishment of vessel networks at the microfluidic platform and in MPS is performed by seeding EC (usually HUVEC, or BMEC if BBB model is reconstructing) as well as accessory cells, (i.e., pericytes or astrocytes) within the chamber channels. To provide efficient endothelial proliferation and vessel sprouting, chemical gradient of pro-angiogenic factors (i.e., VEGF) can be settled in the close vicinity to the growing vessel (Sakolish et al., 2016). Dynamic fluid flow mimics the conditions achieved in the real BBB and allows controlling *in vivo*-like vessel barriergenesis. In a case of BMEC, BBB-specific phenotype of the cells is one of the critical factors for proper modeling the barrier or its functional connection with other tissues/organoids within the MPS (Alcendor et al., 2013). Therefore, the following physiological and metabolic parameters of EPC or BMEC should be taken into the consideration in BBB models or MPS: (i) ability to establish functionally competent monolayer with BBB-specific properties (selective permeability, tight junction connections, expression of specific receptors and transporters) reproducible in either static or microfluidic conditions; (ii) high sensitivity to the action of pro-angiogenic stimuli (VEGF, MMP, lactate etc.) produced by surrounding cells; (iii) dynamic changes in cell metabolism upon action of glia-, pericyte-, or neuron-derived signals; (iv) ontogenesis-related changes in cell metabolism; (v) expression of molecules critical for CNS homing and acquisition of BBB-specific phenotype; (vi) ability to support neurogenesis within the neurogenic niches *in vitro*.

Translational prospects of BBB models *in vitro* or BBB-on-chip technologies dictate the urgent interest in selecting the most appropriate EC whose growth and functional activity would be relevant for *in vitro* testing and drug candidates and assessment of BBB disruption in various CNS disorders. Thus, application of EPC would be beneficial for the controlled growth of brain microvessel-like structures in the conditions close to the BBB microenvironment in order to facilitate further studies of NVU/BBB or cerebrovascular (patho)physiology (Cucullo et al., 2013), particularly, at early stages of ontogenesis. Also, EPC would overcome the problem of human brain microvascular cells availability for BBB modeling or MPS *in vitro*. Currently, few BBB models *in vitro* utilize human endothelial cells, and this problem hampers progress in preclinical pharmacological studies (Pamies et al., 2014). Human induced pluripotent stem cells are tested as a source of NVU components not only in the static BBB models (Lippmann et al., 2012) but also for the microfluidics-based technological solutions (Brown et al., 2015). Microfluidic approach might be also used for specific and efficient capture of EPC from the peripheral blood as it was demonstrated in cardiovascular bioengineering (Plouffe et al., 2009; Lin et al., 2015a). Then, development of “user friendly” protocols for *in vitro* differentiation of EPC obtained from the bone marrow or peripheral blood into BMEC, and for the establishment of NVU microenvironment supporting EPC integration in the growing microvessels would give us an opportunity to establish fully functioning endothelial layer with BBB characteristics. Solving these technological questions would provide novel engineering approaches toward controlling brain developmental and pathological angiogenesis, improved microfluidically-supported BBB models and/or brain multicompartment MPS suitable for translational studies in neurology and neuropharmacology.

## Conclusion

Metabolic and functional plasticity of EPC controls their timely recruitment, precise homing to the brain microvessels, and efficient support of brain angiogenesis. Being under the control of numerous regulatory factors, EPC serve as a source of BMEC with BBB specific characteristics in the developing brain. In the adult brain, EPC contribute (directly or as a source and carrier of pro-angiogenic factors) to BBB re-endothelialization upon injury or to the cerebral angiogenesis associated with physiological conditions (i.e., activity-induced neurogenesis) or pathological conditions (i.e., tumor progression). A key challenge in the field of EPC physiology is the current shortage of the knowledge on the most efficient ways to manipulating their activity both *in vitro* and *in vivo*. However, solving this problem would be beneficial for the therapy of cerebrovascular diseases, brain trauma, neurodegeneration, brain tumors, and neuroinfection. Metabolic activity and functionality of EPC differs from those of BMEC and is tightly regulated by numerous systemic and local factors at all the steps of EPC development. Accumulating evidence suggests that further progress in studying BBB (patho)physiology, establishment of new therapeutic methods for reversible and controlled BBB opening, or designing the new *in vitro* assays for drug development (BBB models or microphysiological constructions) critically depends on deciphering molecular and biochemical mechanisms underlying EPC functional role in the developmental, pathological, and plasticity-driven cerebral angiogenesis.

## Author contributions

NM Drafting the work for important intellectual content; YK Substantial contributions to the conception or design of the work; VS Final approval of the version to be published; AM Drafting the work for important intellectual content; ANS Substantial contributions to the conception or design of the work; YP Substantial contributions to the conception or design of the work; EB Drafting the work for important intellectual content; ABS Substantial contributions to the conception or design of the work, final approval of the version to be published.

### Conflict of interest statement

The authors declare that the research was conducted in the absence of any commercial or financial relationships that could be construed as a potential conflict of interest.

## Abbreviations

Ado: adenosine
ADP: adenosine diphosphate
AMP: adenosine monophosphate
AMPK: AMP-activated protein kinase
ATP: adenosine triphosphate
BBB: blood-brain barrier
BMEC: brain microvessel endothelial cell
BMSC: bone marrow stromal cell
cAMP: cyclic adenosine monophosphate
CD: cluster of differentiation
CNS: central nervous system
CSF: colony-stimulating factor
CXCL12: chemokine (C-X-C-motif) ligand 12
CXCR4: C-X-C chemokine receptor 4
DLL4: delta-like ligand 4
E: day of embryonic development
EC: endothelial cell
EMMPRIN: extracellular matrix metalloproteinase inducer
eNOS: endothelial nitric oxide synthase
EPC: endothelial progenitor cell
Epo: erythropoietin
FA: fatty acid
GLUT: glucose transporter
HIF-1: hypoxia inducible factor-1
HSC: hematopoietic stem cell
IGF: insulin-like growth factor
LDH: lactate dehydrogenase
MCT: monocarboxylate transporter
MIF: macrophage migration inhibitory factor
MMP: matrix metalloproteinase
MPS: microphysiological system
MSC: mesenchymal stem cell
NAD+: nicotinamide adenine dinucleotide
NADH: nicotinamide adenine dinucleotide reduced
NAMPT: nicotinamide phosphorybosyltransferase
NG2: NG2 chondroitin sulfate proteoglycan
NICD: Notch-protein intracellular cytoplasmic domain
NO: nitric oxide
NVU: neurovascular unit
OXPHOS: oxidative phosphorylation
P: day of postnatal development
PDGF: platelet-derived growth factor
PGC-1α: peroxisome proliferator-activated receptor γ coactivator 1α
PPARγ: peroxisome proliferator-activated receptor γ
PPP: pentose phosphate pathway
RGS5: regulator of G-protein signaling 5
ROS: reactive oxygen species
SIRT: sirtuin
SDF-1: stromal cell-derived factor-1
TCA: tricarboxylic acid cycle
TfR: transferrin receptor
TGFβ: transforming growth factor β
TNFα: tumor necrosis factor α
VCAM-1: vascular cell adhesion molecule-1
VEGF: vascular endothelial growth factor
VEGFR: vascular endothelial growth factor receptor
VLA-4: very late antigen-4
vWF: von Willebrand factor
Wnt: Wnt-signaling pathway.
